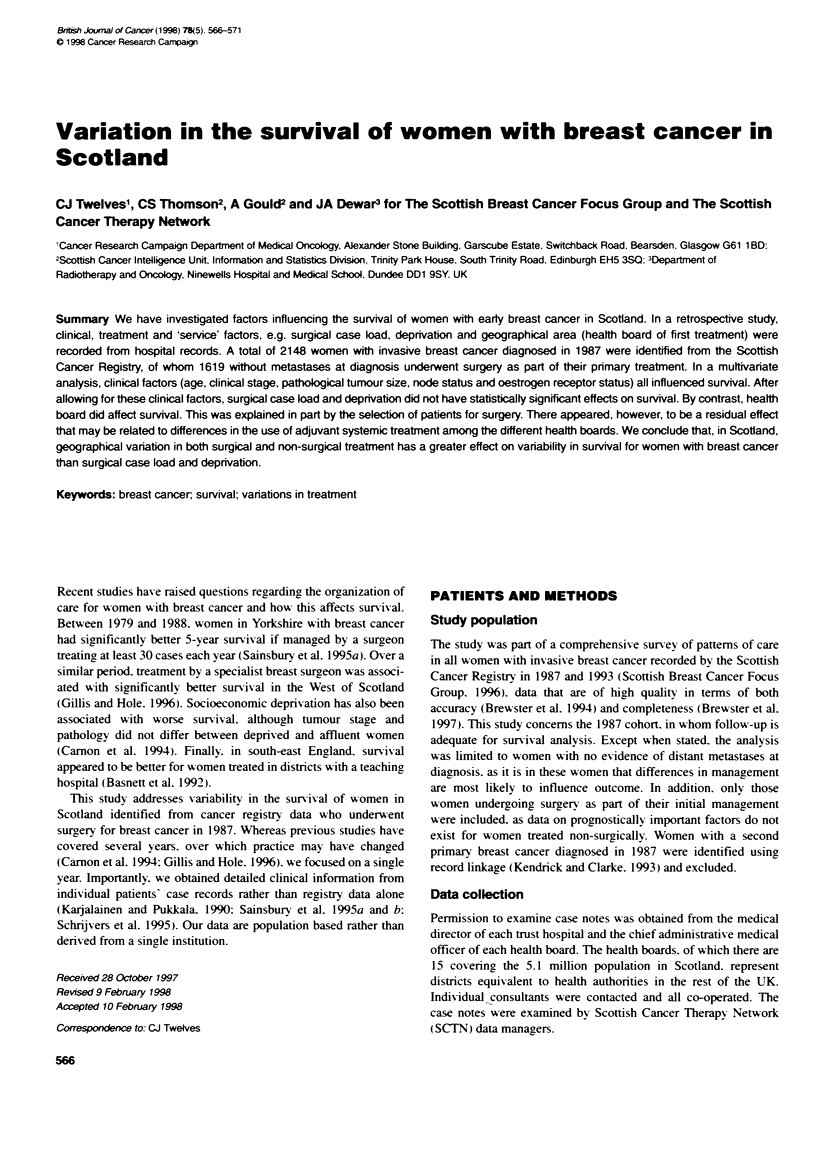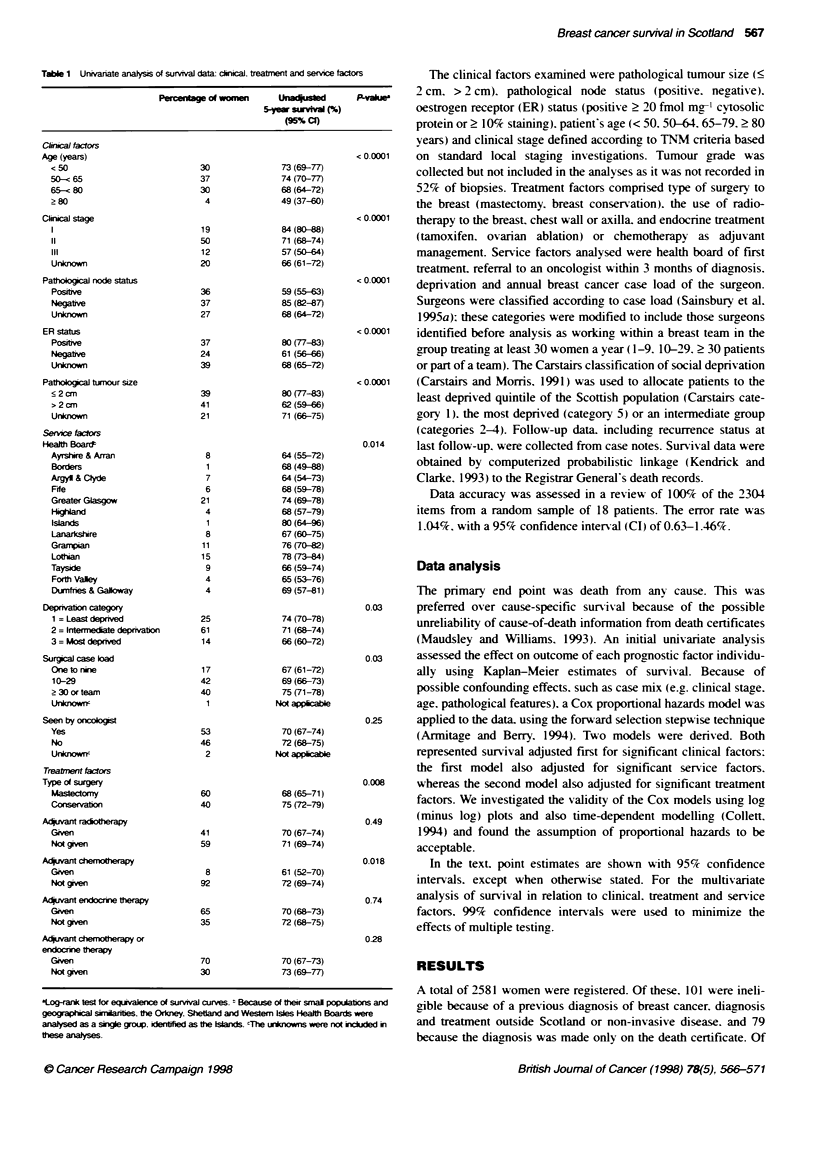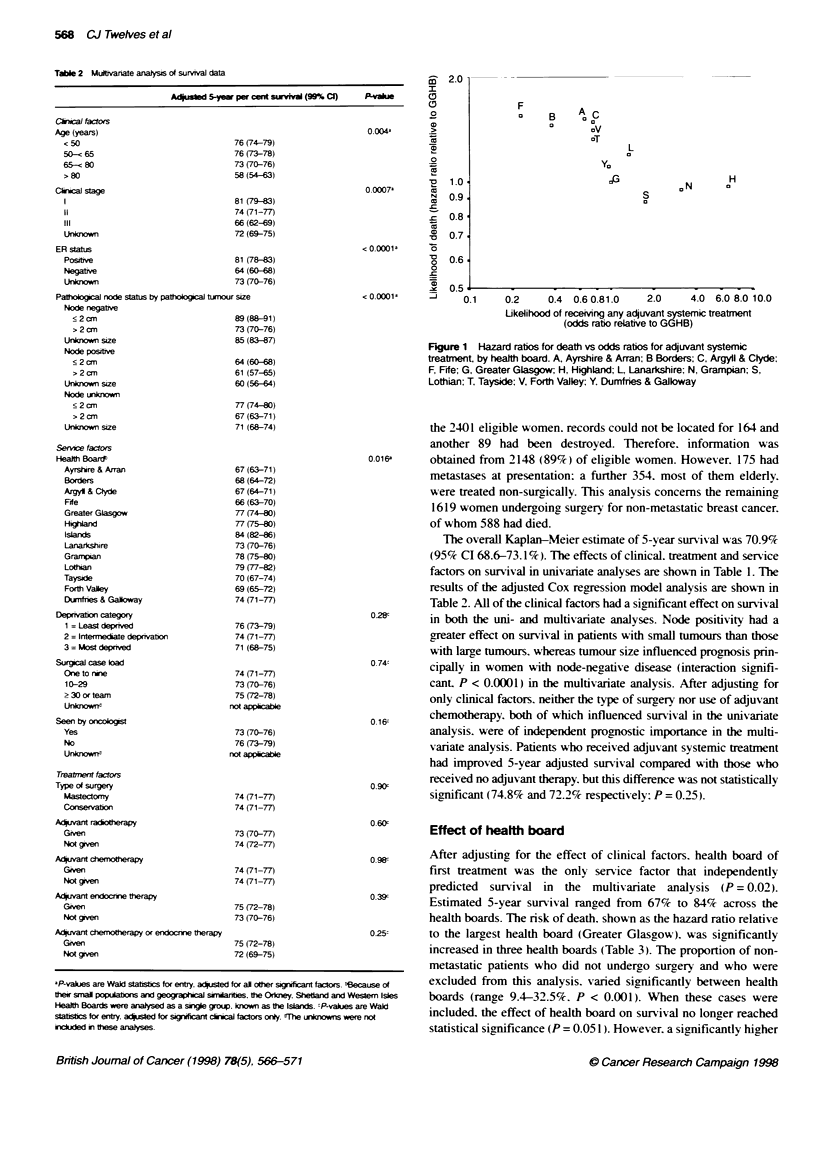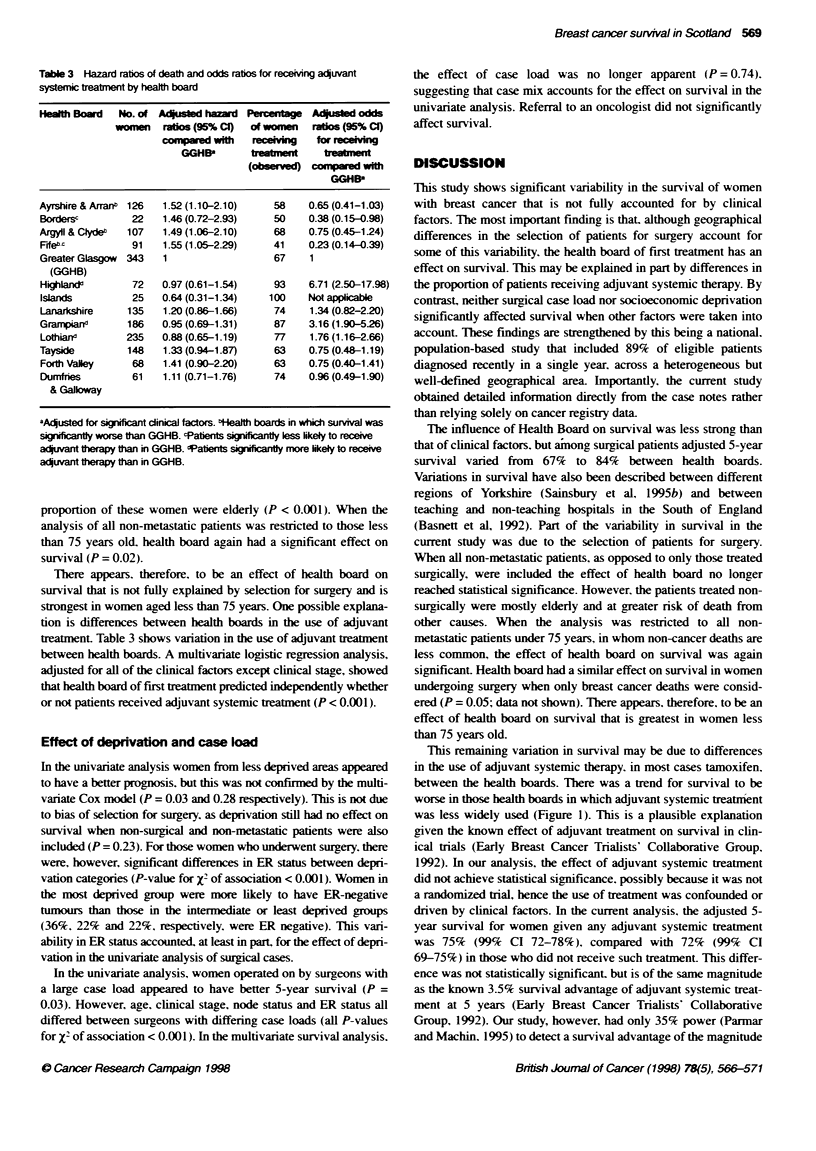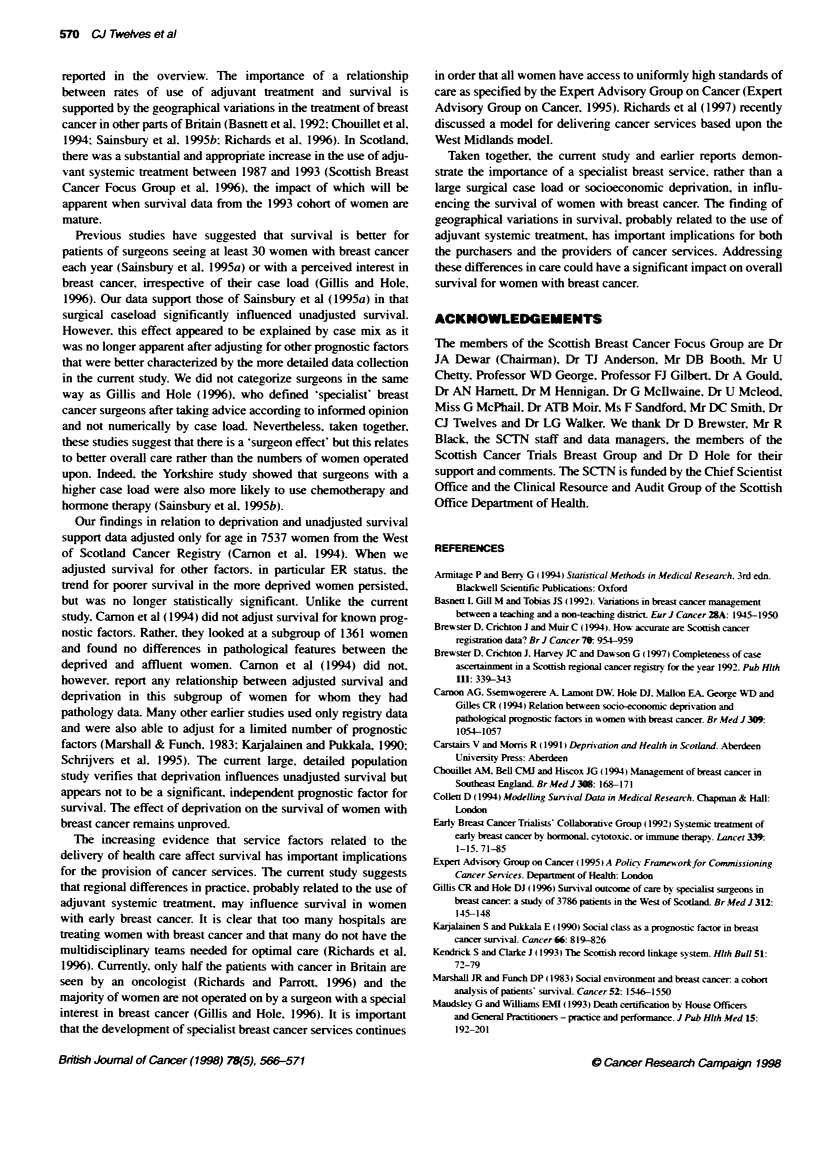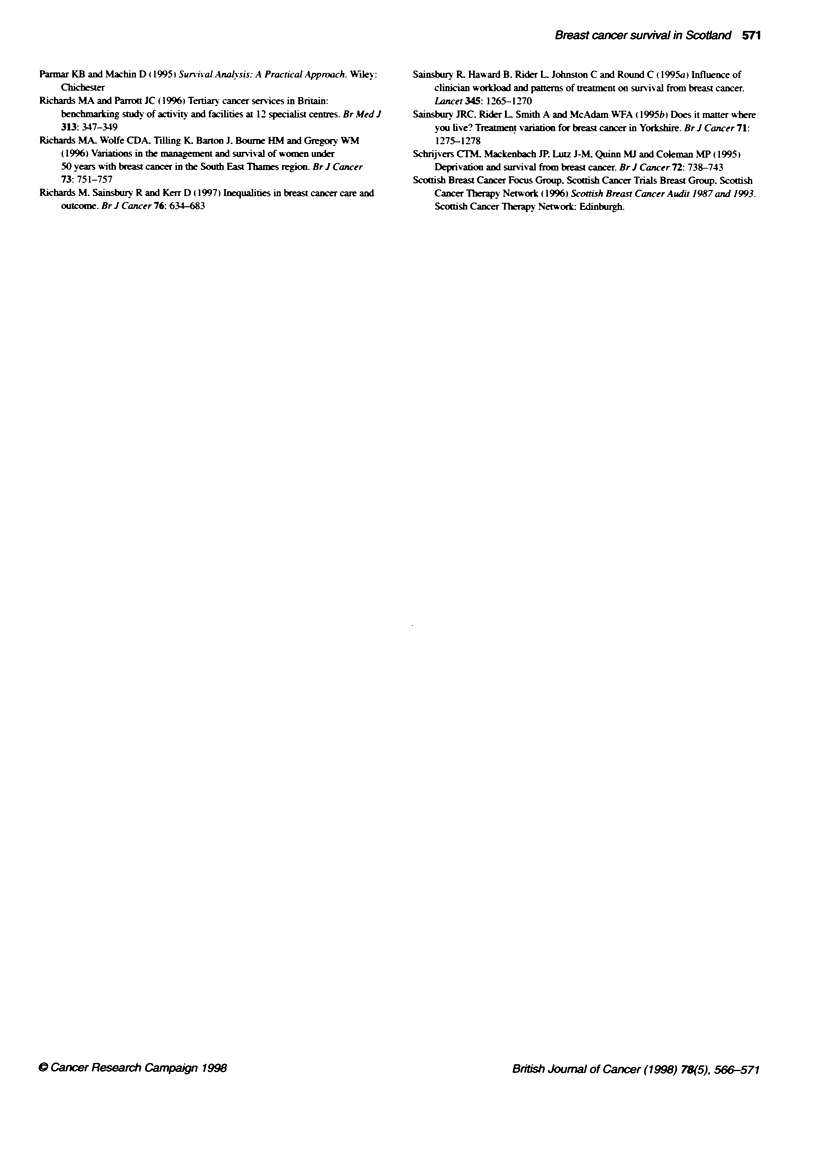# Variation in the survival of women with breast cancer in Scotland. The Scottish Breast Cancer Focus Group and The Scottish Cancer Therapy Network.

**DOI:** 10.1038/bjc.1998.541

**Published:** 1998-09

**Authors:** C. J. Twelves, C. S. Thomson, A. Gould, J. A. Dewar

**Affiliations:** Cancer Research Campaign Department of Medical Oncology, Bearsden, Glasgow.

## Abstract

We have investigated factors influencing the survival of women with early breast cancer in Scotland. In a retrospective study, clinical, treatment and 'service' factors, e.g. surgical case load, deprivation and geographical area (health board of first treatment) were recorded from hospital records. A total of 2148 women with invasive breast cancer diagnosed in 1987 were identified from the Scottish Cancer Registry, of whom 1619 without metastases at diagnosis underwent surgery as part of their primary treatment. In a multivariate analysis, clinical factors (age, clinical stage, pathological tumour size, node status and oestrogen receptor status) all influenced survival. After allowing for these clinical factors, surgical case load and deprivation did not have statistically significant effects on survival. By contrast, health board did affect survival. This was explained in part by the selection of patients for surgery. There appeared, however, to be a residual effect that may be related to differences in the use of adjuvant systemic treatment among the different health boards. We conclude that, in Scotland, geographical variation in both surgical and non-surgical treatment has a greater effect on variability in survival for women with breast cancer than surgical case load and deprivation.


British Journal of Cancer (1998) 78(5), 566–571
© 1998 Cancer Research Campaign

### Variation in the survival of women with breast cancer in Scotland

**CJ Twelves[1], CS Thomson[2], A Gould[2] and JA Dewar[3] for The Scottish Breast Cancer Focus Group and The Scottish Cancer Therapy Network**

[1]Cancer Research Campaign Department of Medical Oncology, Alexander Stone Building, Garscube Estate, Switchback Road, Bearsden, Glasgow G61 1BD; [2]Scottish Cancer Intelligence Unit, Information and Statistics Division, Trinity Park House, South Trinity Road, Edinburgh EH5 3SQ; [3]Department of Radiotherapy and Oncology, Ninewells Hospital and Medical School, Dundee DD1 9SY, UK

**Summary** We have investigated factors influencing the survival of women with early breast cancer in Scotland. In a retrospective study, clinical, treatment and 'service' factors, e.g. surgical case load, deprivation and geographical area (health board of first treatment) were recorded from hospital records. A total of 2148 women with invasive breast cancer diagnosed in 1987 were identified from the Scottish Cancer Registry, of whom 1619 without metastases at diagnosis underwent surgery as part of their primary treatment. In a multivariate analysis, clinical factors (age, clinical stage, pathological tumour size, node status and oestrogen receptor status) all influenced survival. After allowing for these clinical factors, surgical case load and deprivation did not have statistically significant effects on survival. By contrast, health board did affect survival. This was explained in part by the selection of patients for surgery. There appeared, however, to be a residual effect that may be related to differences in the use of adjuvant systemic treatment among the different health boards. We conclude that, in Scotland, geographical variation in both surgical and non-surgical treatment has a greater effect on variability in survival for women with breast cancer than surgical case load and deprivation.

**Keywords:** breast cancer; survival; variations in treatment

Recent studies have raised questions regarding the organization of care for women with breast cancer and how this affects survival. Between 1979 and 1988, women in Yorkshire with breast cancer had significantly better 5-year survival if managed by a surgeon treating at least 30 cases each year (Sainsbury et al, 1995*a*). Over a similar period, treatment by a specialist breast surgeon was associated with significantly better survival in the West of Scotland (Gillis and Hole, 1996). Socioeconomic deprivation has also been associated with worse survival, although tumour stage and pathology did not differ between deprived and affluent women (Carnon et al, 1994). Finally, in south-east England, survival appeared to be better for women treated in districts with a teaching hospital (Basnett et al, 1992).

This study addresses variability in the survival of women in Scotland identified from cancer registry data who underwent surgery for breast cancer in 1987. Whereas previous studies have covered several years, over which practice may have changed (Carnon et al, 1994; Gillis and Hole, 1996), we focused on a single year. Importantly, we obtained detailed clinical information from individual patients' case records rather than registry data alone (Karjalainen and Pukkala, 1990; Sainsbury et al, 1995*a* and *b*; Schrijvers et al, 1995). Our data are population based rather than derived from a single institution.

*Received 28 October 1997*
*Revised 9 February 1998*
*Accepted 10 February 1998*

*Correspondence to:* CJ Twelves

### PATIENTS AND METHODS

### Study population

The study was part of a comprehensive survey of patterns of care in all women with invasive breast cancer recorded by the Scottish Cancer Registry in 1987 and 1993 (Scottish Breast Cancer Focus Group, 1996), data that are of high quality in terms of both accuracy (Brewster et al, 1994) and completeness (Brewster et al, 1997). This study concerns the 1987 cohort, in whom follow-up is adequate for survival analysis. Except when stated, the analysis was limited to women with no evidence of distant metastases at diagnosis, as it is in these women that differences in management are most likely to influence outcome. In addition, only those women undergoing surgery as part of their initial management were included, as data on prognostically important factors do not exist for women treated non-surgically. Women with a second primary breast cancer diagnosed in 1987 were identified using record linkage (Kendrick and Clarke, 1993) and excluded.

### Data collection

Permission to examine case notes was obtained from the medical director of each trust hospital and the chief administrative medical officer of each health board. The health boards, of which there are 15 covering the 5.1 million population in Scotland, represent districts equivalent to health authorities in the rest of the UK. Individual consultants were contacted and all co-operated. The case notes were examined by Scottish Cancer Therapy Network (SCTN) data managers.

**Table 1** Univariate analysis of survival data: clinical, treatment and service factors

| | Percentage of women | Unadjusted 5-year survival (%) (95% CI) | P-value[a] |
|---|---|---|---|
| *Clinical factors* | | | |
| Age (years) | | | < 0.0001 |
| < 50 | 30 | 73 (69–77) | |
| 50–< 65 | 37 | 74 (70–77) | |
| 65–< 80 | 30 | 68 (64–72) | |
| ≥ 80 | 4 | 49 (37–60) | |
| Clinical stage | | | < 0.0001 |
| I | 19 | 84 (80–88) | |
| II | 50 | 71 (68–74) | |
| III | 12 | 57 (50–64) | |
| Unknown | 20 | 66 (61–72) | |
| Pathological node status | | | < 0.0001 |
| Positive | 36 | 59 (55–63) | |
| Negative | 37 | 85 (82–87) | |
| Unknown | 27 | 68 (64–72) | |
| ER status | | | < 0.0001 |
| Positive | 37 | 80 (77–83) | |
| Negative | 24 | 61 (56–66) | |
| Unknown | 39 | 68 (65–72) | |
| Pathological tumour size | | | < 0.0001 |
| ≤ 2 cm | 39 | 80 (77–83) | |
| > 2 cm | 41 | 62 (59–66) | |
| Unknown | 21 | 71 (66–75) | |
| *Service factors* | | | |
| Health Board[b] | | | 0.014 |
| Ayrshire & Arran | 8 | 64 (55–72) | |
| Borders | 1 | 68 (49–88) | |
| Argyll & Clyde | 7 | 64 (54–73) | |
| Fife | 6 | 68 (59–78) | |
| Greater Glasgow | 21 | 74 (69–78) | |
| Highland | 4 | 68 (57–79) | |
| Islands | 1 | 80 (64–96) | |
| Lanarkshire | 8 | 67 (60–75) | |
| Grampian | 11 | 76 (70–82) | |
| Lothian | 15 | 78 (73–84) | |
| Tayside | 9 | 66 (59–74) | |
| Forth Valley | 4 | 65 (53–76) | |
| Dumfries & Galloway | 4 | 69 (57–81) | |
| Deprivation category | | | 0.03 |
| 1 = Least deprived | 25 | 74 (70–78) | |
| 2 = Intermediate deprivation | 61 | 71 (68–74) | |
| 3 = Most deprived | 14 | 66 (60–72) | |
| Surgical case load | | | 0.03 |
| One to nine | 17 | 67 (61–72) | |
| 10–29 | 42 | 69 (66–73) | |
| ≥ 30 or team | 40 | 75 (71–78) | |
| Unknown[c] | 1 | Not applicable | |
| Seen by oncologist | | | 0.25 |
| Yes | 53 | 70 (67–74) | |
| No | 46 | 72 (68–75) | |
| Unknown[c] | 2 | Not applicable | |
| *Treatment factors* | | | |
| Type of surgery | | | 0.008 |
| Mastectomy | 60 | 68 (65–71) | |
| Conservation | 40 | 75 (72–79) | |
| Adjuvant radiotherapy | | | 0.49 |
| Given | 41 | 70 (67–74) | |
| Not given | 59 | 71 (69–74) | |
| Adjuvant chemotherapy | | | 0.018 |
| Given | 8 | 61 (52–70) | |
| Not given | 92 | 72 (69–74) | |
| Adjuvant endocrine therapy | | | 0.74 |
| Given | 65 | 70 (68–73) | |
| Not given | 35 | 72 (68–75) | |
| Adjuvant chemotherapy or endocrine therapy | | | 0.28 |
| Given | 70 | 70 (67–73) | |
| Not given | 30 | 73 (69–77) | |

[a]Log-rank test for equivalence of survival curves. [b]Because of their small populations and geographical similarities, the Orkney, Shetland and Western Isles Health Boards were analysed as a single group, identified as the Islands. [c]The unknowns were not included in these analyses.

The clinical factors examined were pathological tumour size (≤ 2 cm, > 2 cm), pathological node status (positive, negative), oestrogen receptor (ER) status (positive ≥ 20 fmol mg$^{-1}$ cytosolic protein or ≥ 10% staining), patient's age (< 50, 50–64, 65–79, ≥ 80 years) and clinical stage defined according to TNM criteria based on standard local staging investigations. Tumour grade was collected but not included in the analyses as it was not recorded in 52% of biopsies. Treatment factors comprised type of surgery to the breast (mastectomy, breast conservation), the use of radiotherapy to the breast, chest wall or axilla, and endocrine treatment (tamoxifen, ovarian ablation) or chemotherapy as adjuvant management. Service factors analysed were health board of first treatment, referral to an oncologist within 3 months of diagnosis, deprivation and annual breast cancer case load of the surgeon. Surgeons were classified according to case load (Sainsbury et al, 1995*a*): these categories were modified to include those surgeons identified before analysis as working within a breast team in the group treating at least 30 women a year (1–9, 10–29, ≥ 30 patients or part of a team). The Carstairs classification of social deprivation (Carstairs and Morris, 1991) was used to allocate patients to the least deprived quintile of the Scottish population (Carstairs category 1), the most deprived (category 5) or an intermediate group (categories 2–4). Follow-up data, including recurrence status at last follow-up, were collected from case notes. Survival data were obtained by computerized probabilistic linkage (Kendrick and Clarke, 1993) to the Registrar General's death records.

Data accuracy was assessed in a review of 100% of the 2304 items from a random sample of 18 patients. The error rate was 1.04%, with a 95% confidence interval (CI) of 0.63–1.46%.

### Data analysis

The primary end point was death from any cause. This was preferred over cause-specific survival because of the possible unreliability of cause-of-death information from death certificates (Maudsley and Williams, 1993). An initial univariate analysis assessed the effect on outcome of each prognostic factor individually using Kaplan–Meier estimates of survival. Because of possible confounding effects, such as case mix (e.g. clinical stage, age, pathological features), a Cox proportional hazards model was applied to the data, using the forward selection stepwise technique (Armitage and Berry, 1994). Two models were derived. Both represented survival adjusted first for significant clinical factors; the first model also adjusted for significant service factors, whereas the second model also adjusted for significant treatment factors. We investigated the validity of the Cox models using log (minus log) plots and also time-dependent modelling (Collett, 1994) and found the assumption of proportional hazards to be acceptable.

In the text, point estimates are shown with 95% confidence intervals, except when otherwise stated. For the multivariate analysis of survival in relation to clinical, treatment and service factors, 99% confidence intervals were used to minimize the effects of multiple testing.

### RESULTS

A total of 2581 women were registered. Of these, 101 were ineligible because of a previous diagnosis of breast cancer, diagnosis and treatment outside Scotland or non-invasive disease, and 79 because the diagnosis was made only on the death certificate. Of

© Cancer Research Campaign 1998

**Table 2** Multivariate analysis of survival data

| | Adjusted 5-year per cent survival (99% CI) | P-value |
|---|---|---|
| *Clinical factors* | | |
| Age (years) | | 0.004[a] |
| < 50 | 76 (74–79) | |
| 50–< 65 | 76 (73–78) | |
| 65–< 80 | 73 (70–76) | |
| > 80 | 58 (54–63) | |
| Clinical stage | | 0.0007[a] |
| I | 81 (79–83) | |
| II | 74 (71–77) | |
| III | 66 (62–69) | |
| Unknown | 72 (69–75) | |
| ER status | | < 0.0001[a] |
| Positive | 81 (78–83) | |
| Negative | 64 (60–68) | |
| Unknown | 73 (70–76) | |
| Pathological node status by pathological tumour size | | < 0.0001[a] |
| Node negative | | |
| ≤ 2 cm | 89 (88–91) | |
| > 2 cm | 73 (70–76) | |
| Unknown size | 85 (83–87) | |
| Node positive | | |
| ≤ 2 cm | 64 (60–68) | |
| > 2 cm | 61 (57–65) | |
| Unknown size | 60 (56–64) | |
| Node unknown | | |
| ≤ 2 cm | 77 (74–80) | |
| > 2 cm | 67 (63–71) | |
| Unknown size | 71 (68–74) | |
| *Service factors* | | |
| Health Board[b] | | 0.016[a] |
| Ayrshire & Arran | 67 (63–71) | |
| Borders | 68 (64–72) | |
| Argyll & Clyde | 67 (64–71) | |
| Fife | 66 (63–70) | |
| Greater Glasgow | 77 (74–80) | |
| Highland | 77 (75–80) | |
| Islands | 84 (82–86) | |
| Lanarkshire | 73 (70–76) | |
| Grampian | 78 (75–80) | |
| Lothian | 79 (77–82) | |
| Tayside | 70 (67–74) | |
| Forth Valley | 69 (65–72) | |
| Dumfries & Galloway | 74 (71–77) | |
| Deprivation category | | 0.28[c] |
| 1 = Least deprived | 76 (73–79) | |
| 2 = Intermediate deprivation | 74 (71–77) | |
| 3 = Most deprived | 71 (68–75) | |
| Surgical case load | | 0.74[c] |
| One to nine | 74 (71–77) | |
| 10–29 | 73 (70–76) | |
| ≥ 30 or team | 75 (72–78) | |
| Unknown[d] | not applicable | |
| Seen by oncologist | | 0.16[c] |
| Yes | 73 (70–76) | |
| No | 76 (73–79) | |
| Unknown[d] | not applicable | |
| *Treatment factors* | | |
| Type of surgery | | 0.90[c] |
| Mastectomy | 74 (71–77) | |
| Conservation | 74 (71–77) | |
| Adjuvant radiotherapy | | 0.60[c] |
| Given | 73 (70–77) | |
| Not given | 74 (72–77) | |
| Adjuvant chemotherapy | | 0.98[c] |
| Given | 74 (71–77) | |
| Not given | 74 (71–77) | |
| Adjuvant endocrine therapy | | 0.39[c] |
| Given | 75 (72–78) | |
| Not given | 73 (70–76) | |
| Adjuvant chemotherapy or endocrine therapy | | 0.25[c] |
| Given | 75 (72–78) | |
| Not given | 72 (69–75) | |

[a]P-values are Wald statistics for entry, adjusted for all other significant factors. [b]Because of their small populations and geographical similarities, the Orkney, Shetland and Western Isles Health Boards were analysed as a single group, known as the Islands. [c]P-values are Wald statistics for entry, adjusted for significant clinical factors only. [d]The unknowns were not included in these analyses.

**Figure 1** Hazard ratios for death vs odds ratios for adjuvant systemic treatment, by health board. A, Ayrshire & Arran; B Borders; C, Argyll & Clyde; F, Fife; G, Greater Glasgow; H, Highland; L, Lanarkshire; N, Grampian; S, Lothian; T, Tayside; V, Forth Valley; Y, Dumfries & Galloway the 2401 eligible women, records could not be located for 164 and another 89 had been destroyed. Therefore, information was obtained from 2148 (89%) of eligible women. However, 175 had metastases at presentation; a further 354, most of them elderly, were treated non-surgically. This analysis concerns the remaining 1619 women undergoing surgery for non-metastatic breast cancer, of whom 588 had died.

The overall Kaplan–Meier estimate of 5-year survival was 70.9% (95% CI 68.6–73.1%). The effects of clinical, treatment and service factors on survival in univariate analyses are shown in Table 1. The results of the adjusted Cox regression model analysis are shown in Table 2. All of the clinical factors had a significant effect on survival in both the uni- and multivariate analyses. Node positivity had a greater effect on survival in patients with small tumours than those with large tumours, whereas tumour size influenced prognosis principally in women with node-negative disease (interaction significant, $P < 0.0001$) in the multivariate analysis. After adjusting for only clinical factors, neither the type of surgery nor use of adjuvant chemotherapy, both of which influenced survival in the univariate analysis, were of independent prognostic importance in the multivariate analysis. Patients who received adjuvant systemic treatment had improved 5-year adjusted survival compared with those who received no adjuvant therapy, but this difference was not statistically significant (74.8% and 72.2% respectively; $P = 0.25$).

### Effect of health board

After adjusting for the effect of clinical factors, health board of first treatment was the only service factor that independently predicted survival in the multivariate analysis ($P = 0.02$). Estimated 5-year survival ranged from 67% to 84% across the health boards. The risk of death, shown as the hazard ratio relative to the largest health board (Greater Glasgow), was significantly increased in three health boards (Table 3). The proportion of non-metastatic patients who did not undergo surgery and who were excluded from this analysis, varied significantly between health boards (range 9.4–32.5%, $P < 0.001$). When these cases were included, the effect of health board on survival no longer reached statistical significance ($P = 0.051$). However, a significantly higher

© Cancer Research Campaign 1998

**Table 3** Hazard ratios of death and odds ratios for receiving adjuvant systemic treatment by health board

| Health Board | No. of women | Adjusted hazard ratios (95% CI) compared with GGHB[a] | Percentage of women receiving treatment (observed) | Adjusted odds ratios (95% CI) for receiving treatment compared with GGHB[a] |
|---|---|---|---|---|
| Ayrshire & Arran[b] | 126 | 1.52 (1.10–2.10) | 58 | 0.65 (0.41–1.03) |
| Borders[c] | 22 | 1.46 (0.72–2.93) | 50 | 0.38 (0.15–0.98) |
| Argyll & Clyde[b] | 107 | 1.49 (1.06–2.10) | 68 | 0.75 (0.45–1.24) |
| Fife[b,c] | 91 | 1.55 (1.05–2.29) | 41 | 0.23 (0.14–0.39) |
| Greater Glasgow (GGHB) | 343 | 1 | 67 | 1 |
| Highland[d] | 72 | 0.97 (0.61–1.54) | 93 | 6.71 (2.50–17.98) |
| Islands | 25 | 0.64 (0.31–1.34) | 100 | Not applicable |
| Lanarkshire | 135 | 1.20 (0.86–1.66) | 74 | 1.34 (0.82–2.20) |
| Grampian[d] | 186 | 0.95 (0.69–1.31) | 87 | 3.16 (1.90–5.26) |
| Lothian[d] | 235 | 0.88 (0.65–1.19) | 77 | 1.76 (1.16–2.66) |
| Tayside | 148 | 1.33 (0.94–1.87) | 63 | 0.75 (0.48–1.19) |
| Forth Valley | 68 | 1.41 (0.90–2.20) | 63 | 0.75 (0.40–1.41) |
| Dumfries & Galloway | 61 | 1.11 (0.71–1.76) | 74 | 0.96 (0.49–1.90) |

[a]Adjusted for significant clinical factors. [b]Health boards in which survival was significantly worse than GGHB. [c]Patients significantly less likely to receive adjuvant therapy than in GGHB. [d]Patients significantly more likely to receive adjuvant therapy than in GGHB.

proportion of these women were elderly ($P < 0.001$). When the analysis of all non-metastatic patients was restricted to those less than 75 years old, health board again had a significant effect on survival ($P = 0.02$).

There appears, therefore, to be an effect of health board on survival that is not fully explained by selection for surgery and is strongest in women aged less than 75 years. One possible explanation is differences between health boards in the use of adjuvant treatment. Table 3 shows variation in the use of adjuvant treatment between health boards. A multivariate logistic regression analysis, adjusted for all of the clinical factors except clinical stage, showed that health board of first treatment predicted independently whether or not patients received adjuvant systemic treatment ($P < 0.001$).

### Effect of deprivation and case load

In the univariate analysis women from less deprived areas appeared to have a better prognosis, but this was not confirmed by the multivariate Cox model ($P = 0.03$ and 0.28 respectively). This is not due to bias of selection for surgery, as deprivation still had no effect on survival when non-surgical and non-metastatic patients were also included ($P = 0.23$). For those women who underwent surgery, there were, however, significant differences in ER status between deprivation categories ($P$-value for $\chi^2$ of association < 0.001). Women in the most deprived group were more likely to have ER-negative tumours than those in the intermediate or least deprived groups (36%, 22% and 22%, respectively, were ER negative). This variability in ER status accounted, at least in part, for the effect of deprivation in the univariate analysis of surgical cases.

In the univariate analysis, women operated on by surgeons with a large case load appeared to have better 5-year survival ($P$ = 0.03). However, age, clinical stage, node status and ER status all differed between surgeons with differing case loads (all $P$-values for $\chi^2$ of association < 0.001). In the multivariate survival analysis, the effect of case load was no longer apparent ($P = 0.74$), suggesting that case mix accounts for the effect on survival in the univariate analysis. Referral to an oncologist did not significantly affect survival.

### DISCUSSION

This study shows significant variability in the survival of women with breast cancer that is not fully accounted for by clinical factors. The most important finding is that, although geographical differences in the selection of patients for surgery account for some of this variability, the health board of first treatment has an effect on survival. This may be explained in part by differences in the proportion of patients receiving adjuvant systemic therapy. By contrast, neither surgical case load nor socioeconomic deprivation significantly affected survival when other factors were taken into account. These findings are strengthened by this being a national, population-based study that included 89% of eligible patients diagnosed recently in a single year, across a heterogeneous but well-defined geographical area. Importantly, the current study obtained detailed information directly from the case notes rather than relying solely on cancer registry data.

The influence of Health Board on survival was less strong than that of clinical factors, but among surgical patients adjusted 5-year survival varied from 67% to 84% between health boards. Variations in survival have also been described between different regions of Yorkshire (Sainsbury et al, 1995*b*) and between teaching and non-teaching hospitals in the South of England (Basnett et al, 1992). Part of the variability in survival in the current study was due to the selection of patients for surgery. When all non-metastatic patients, as opposed to only those treated surgically, were included the effect of health board no longer reached statistical significance. However, the patients treated non-surgically were mostly elderly and at greater risk of death from other causes. When the analysis was restricted to all non-metastatic patients under 75 years, in whom non-cancer deaths are less common, the effect of health board on survival was again significant. Health board had a similar effect on survival in women undergoing surgery when only breast cancer deaths were considered ($P = 0.05$; data not shown). There appears, therefore, to be an effect of health board on survival that is greatest in women less than 75 years old.

This remaining variation in survival may be due to differences in the use of adjuvant systemic therapy, in most cases tamoxifen, between the health boards. There was a trend for survival to be worse in those health boards in which adjuvant systemic treatment was less widely used (Figure 1). This is a plausible explanation given the known effect of adjuvant treatment on survival in clinical trials (Early Breast Cancer Trialists' Collaborative Group, 1992). In our analysis, the effect of adjuvant systemic treatment did not achieve statistical significance, possibly because it was not a randomized trial, hence the use of treatment was confounded or driven by clinical factors. In the current analysis, the adjusted 5-year survival for women given any adjuvant systemic treatment was 75% (99% CI 72–78%), compared with 72% (99% CI 69–75%) in those who did not receive such treatment. This difference was not statistically significant, but is of the same magnitude as the known 3.5% survival advantage of adjuvant systemic treatment at 5 years (Early Breast Cancer Trialists' Collaborative Group, 1992). Our study, however, had only 35% power (Parmar and Machin, 1995) to detect a survival advantage of the magnitude reported in the overview. The importance of a relationship between rates of use of adjuvant treatment and survival is supported by the geographical variations in the treatment of breast cancer in other parts of Britain (Basnett et al, 1992; Chouillet et al, 1994; Sainsbury et al, 1995*b*; Richards et al, 1996). In Scotland, there was a substantial and appropriate increase in the use of adjuvant systemic treatment between 1987 and 1993 (Scottish Breast Cancer Focus Group et al, 1996), the impact of which will be apparent when survival data from the 1993 cohort of women are mature.

Previous studies have suggested that survival is better for patients of surgeons seeing at least 30 women with breast cancer each year (Sainsbury et al, 1995*a*) or with a perceived interest in breast cancer, irrespective of their case load (Gillis and Hole, 1996). Our data support those of Sainsbury et al (1995*a*) in that surgical caseload significantly influenced unadjusted survival. However, this effect appeared to be explained by case mix as it was no longer apparent after adjusting for other prognostic factors that were better characterized by the more detailed data collection in the current study. We did not categorize surgeons in the same way as Gillis and Hole (1996), who defined 'specialist' breast cancer surgeons after taking advice according to informed opinion and not numerically by case load. Nevertheless, taken together, these studies suggest that there is a 'surgeon effect' but this relates to better overall care rather than the numbers of women operated upon. Indeed, the Yorkshire study showed that surgeons with a higher case load were also more likely to use chemotherapy and hormone therapy (Sainsbury et al, 1995*b*).

Our findings in relation to deprivation and unadjusted survival support data adjusted only for age in 7537 women from the West of Scotland Cancer Registry (Carnon et al, 1994). When we adjusted survival for other factors, in particular ER status, the trend for poorer survival in the more deprived women persisted, but was no longer statistically significant. Unlike the current study, Carnon et al (1994) did not adjust survival for known prognostic factors. Rather, they looked at a subgroup of 1361 women and found no differences in pathological features between the deprived and affluent women. Carnon et al (1994) did not, however, report any relationship between adjusted survival and deprivation in this subgroup of women for whom they had pathology data. Many other earlier studies used only registry data and were also able to adjust for a limited number of prognostic factors (Marshall & Funch, 1983; Karjalainen and Pukkala, 1990; Schrijvers et al, 1995). The current large, detailed population study verifies that deprivation influences unadjusted survival but appears not to be a significant, independent prognostic factor for survival. The effect of deprivation on the survival of women with breast cancer remains unproved.

The increasing evidence that service factors related to the delivery of health care affect survival has important implications for the provision of cancer services. The current study suggests that regional differences in practice, probably related to the use of adjuvant systemic treatment, may influence survival in women with early breast cancer. It is clear that too many hospitals are treating women with breast cancer and that many do not have the multidisciplinary teams needed for optimal care (Richards et al, 1996). Currently, only half the patients with cancer in Britain are seen by an oncologist (Richards and Parrott, 1996) and the majority of women are not operated on by a surgeon with a special interest in breast cancer (Gillis and Hole, 1996). It is important that the development of specialist breast cancer services continues in order that all women have access to uniformly high standards of care as specified by the Expert Advisory Group on Cancer (Expert Advisory Group on Cancer, 1995). Richards et al (1997) recently discussed a model for delivering cancer services based upon the West Midlands model.

Taken together, the current study and earlier reports demonstrate the importance of a specialist breast service, rather than a large surgical case load or socioeconomic deprivation, in influencing the survival of women with breast cancer. The finding of geographical variations in survival, probably related to the use of adjuvant systemic treatment, has important implications for both the purchasers and the providers of cancer services. Addressing these differences in care could have a significant impact on overall survival for women with breast cancer.

### ACKNOWLEDGEMENTS

The members of the Scottish Breast Cancer Focus Group are Dr JA Dewar (Chairman), Dr TJ Anderson, Mr DB Booth, Mr U Chetty, Professor WD George, Professor FJ Gilbert, Dr A Gould, Dr AN Harnett, Dr M Hennigan, Dr G McIlwaine, Dr U Mcleod, Miss G McPhail, Dr ATB Moir, Ms F Sandford, Mr DC Smith, Dr CJ Twelves and Dr LG Walker. We thank Dr D Brewster, Mr R Black, the SCTN staff and data managers, the members of the Scottish Cancer Trials Breast Group and Dr D Hole for their support and comments. The SCTN is funded by the Chief Scientist Office and the Clinical Resource and Audit Group of the Scottish Office Department of Health.

### REFERENCES

Armitage P and Berry G (1994) *Statistical Methods in Medical Research*, 3rd edn. Blackwell Scientific Publications: Oxford

Basnett I, Gill M and Tobias JS (1992). Variations in breast cancer management between a teaching and a non-teaching district. *Eur J Cancer* **28A**: 1945–1950

Brewster D, Crichton J and Muir C (1994). How accurate are Scottish cancer registration data? *Br J Cancer* **70**: 954–959

Brewster D, Crichton J, Harvey JC and Dawson G (1997) Completeness of case ascertainment in a Scottish regional cancer registry for the year 1992. *Pub Hlth* **111**: 339–343

Carnon AG, Ssemwogerere A, Lamont DW, Hole DJ, Mallon EA, George WD and Gilles CR (1994) Relation between socio-economic deprivation and pathological prognostic factors in women with breast cancer. *Br Med J* **309**: 1054–1057

Carstairs V and Morris R (1991) *Deprivation and Health in Scotland*. Aberdeen University Press: Aberdeen

Chouillet AM, Bell CMJ and Hiscox JG (1994) Management of breast cancer in Southeast England. *Br Med J* **308**: 168–171

Collett D (1994) *Modelling Survival Data in Medical Research*. Chapman & Hall: London

Early Breast Cancer Trialists' Collaborative Group (1992) Systemic treatment of early breast cancer by hormonal, cytotoxic, or immune therapy. *Lancet* **339**: 1–15, 71–85

Expert Advisory Group on Cancer (1995) *A Policy Framework for Commissioning Cancer Services*. Department of Health: London

Gillis CR and Hole DJ (1996) Survival outcome of care by specialist surgeons in breast cancer: a study of 3786 patients in the West of Scotland. *Br Med J* **312**: 145–148

Karjalainen S and Pukkala E (1990) Social class as a prognostic factor in breast cancer survival. *Cancer* **66**: 819–826

Kendrick S and Clarke J (1993) The Scottish record linkage system. *Hlth Bull* **51**: 72–79

Marshall JR and Funch DP (1983) Social environment and breast cancer: a cohort analysis of patients' survival. *Cancer* **52**: 1546–1550

Maudsley G and Williams EMI (1993) Death certification by House Officers and General Practitioners – practice and performance. *J Pub Hlth Med* **15**: 192–201

 © Cancer Research Campaign 1998

Parmar KB and Machin D (1995) *Survival Analysis: A Practical Approach*. Wiley: Chichester

Richards MA and Parrott JC (1996) Tertiary cancer services in Britain: benchmarking study of activity and facilities at 12 specialist centres. *Br Med J* **313**: 347–349

Richards MA, Wolfe CDA, Tilling K, Barton J, Bourne HM and Gregory WM (1996) Variations in the management and survival of women under 50 years with breast cancer in the South East Thames region. *Br J Cancer* **73**: 751–757

Richards M, Sainsbury R and Kerr D (1997) Inequalities in breast cancer care and outcome. *Br J Cancer* **76**: 634–683

Sainsbury R, Haward B, Rider L, Johnston C and Round C (1995*a*) Influence of clinician workload and patterns of treatment on survival from breast cancer. *Lancet* **345**: 1265–1270

Sainsbury JRC, Rider L, Smith A and McAdam WFA (1995*b*) Does it matter where you live? Treatment variation for breast cancer in Yorkshire. *Br J Cancer* **71**: 1275–1278

Schrijvers CTM, Mackenbach JP, Lutz J-M, Quinn MJ and Coleman MP (1995) Deprivation and survival from breast cancer. *Br J Cancer* **72**: 738–743

Scottish Breast Cancer Focus Group, Scottish Cancer Trials Breast Group, Scottish Cancer Therapy Network (1996) *Scottish Breast Cancer Audit 1987 and 1993*. Scottish Cancer Therapy Network: Edinburgh.

© Cancer Research Campaign 1998